# Construction of a High-Expression System in Bacillus through Transcriptomic Profiling and Promoter Engineering

**DOI:** 10.3390/microorganisms8071030

**Published:** 2020-07-12

**Authors:** Cui-Cui Miao, Lin-Li Han, Yan-Bing Lu, Hong Feng

**Affiliations:** 1 Key Laboratory of Molecular Biology and Biotechnology, College of Life Sciences, Sichuan University, 29, Wangjiang Rd, Chengdu 610064, China; meffort@foxmail.com (C.-C.M.); linlihan2019@foxmail.com (L.-L.H.); 18215563276@163.com (Y.-B.L.); 2 Key Laboratory for Bio-Resources and Eco-Environment of Ministry of Education, Sichuan University, 29 Wangjiang Rd, Chengdu 610064, China

**Keywords:** *B. subtilis*, promoter, promoter engineering, recombinant expression, induction

## Abstract

*Bacillus subtilis* is an ideal host for secretion and expression of foreign proteins. The promoter is one of the most important elements to facilitate the high-level production of recombinant protein. To expand the repertoire of strong promoters for biotechnological applications in *Bacillus* species, 14 highly transcribed genes based on transcriptome profiling of *B. pumilus* BA06 were selected and evaluated for their promoter strength in *B. subtilis*. Consequently, a strong promoter P_2069_ was obtained, which could drive the genes encoding alkaline protease (*aprE*) and green fluorescent protein (GFP) to express more efficiency by an increase of 3.65-fold and 18.40-fold in comparison with the control promoter (P_aprE_), respectively. Further, promoter engineering was applied to P_2069_, leading to a mutation promoter (P_2069M_) that could increase GFP expression by 3.67-fold over the wild-type promoter (P_2069_). Moreover, the IPTG-inducible expression systems were constructed using the *lac* operon based on the strong promoters of P_2069_ and P_2069M_, which could work well both in *B. subtilis* and *B. pumilus*. In this study, highly efficient expression system for *Bacillus* was constructed based on transcriptome data and promoter engineering, which provide not only a new option for recombinant expression in *B. subtilis*, but also novel genetic tool for *B. pumilus*.

## 1. Introduction

*Bacillus subtilis*, a rod-shaped Gram-positive soil bacterium, has been used as a model microorganism both in basic research and biotechnological applications for more than a century [1,2,3,4]. It can produce and secrete abundant industrial proteins [5]. Therefore, *B. subtilis* is developed as an expression host for the production of recombinant proteins [6,7,8,9]. The major advantages of *B. subtilis* over the other gene expression hosts include non-biased codon usage, high-cell–density fermentation and protein secretion capability [6,7,8,9,10,11]. Along with availability of the complete genome sequence of *B. subtilis* [12], a number of genetic tools, including various expression vectors, promoters, regulatory elements, and signal peptides, have been developed and characterized [13,14,15,16]. Although a variety of heterologous genes have been expressed successfully in *B. subtilis*, the plasmid instability and lower protein production hindered its application [17]. Thus, efficient production of high-value recombinant proteins in *B. subtilis* remains a major challenge. In order to obtain large amounts of recombinant proteins, it is an important way to use high-copy plasmids and strong promoters. Promoters are important regulatory elements, which facilitate high-level gene expression and recombinant protein production [6,8]. Several approaches have been used to identify novel promoters, including screening of chromosomal DNA fragments [18,19], modification of promoter sequences [20] and construction of tandem promoters [21]. A bioinformatic method for selecting candidate strong promoters was also developed by analyzing the highly transcribed genes based on the transcriptome data [22] because the genome-wide expression levels were largely correlated to the upstream promoter strength [23]. Therefore, transcriptomic data are useful information for screening strong promoters in biotechnological applications [24].

In addition to the native strong promoters, heterologous promoters have been also used in *B. subtilis* to achieve higher levels of gene expression. As early as in 1980s, Lee et al. [25] characterized a series of promoters from the *B. subtilis* bacteriophage SPO1, out which the promoters SPO1-15 and SPO1-16 were proved to be strong constitutive promoters and used to overexpress a heterologous pathway to produce a fine compound (pyridoxine) in *B. subtilis* [26]. Further, the heterologous phage promoter from the other bacteria such as *E. coli* can also be exploited for recombinant protein expression in *B. subtilis* [27].

In recent years, numerous strategies focusing on promoter engineering have been developed and applied to overcome the bottleneck of intrinsic promoters [17]. Preferably, there are two methods for engineering native promoters, which are the tandem promoter strategy [20,21] and semi-rational evolution [28,29]. Transcriptional initiation driven by the bacterial promoters is highly dependent on two conserved sequences in the core promoter, the −10 and the −35 regions. Although these two elements determine transcriptional strength, the spacer regions between them help to fine tune the transcription level [30]. Inspired by this strategy, a study expanded the framework of promoter engineering by using a semi-rationally engineered promoter library for the spacer sequence between the −10 and −35 regions of P_srfA_, and an engineered promoter P_v1_ was screened out from the mutation promoter library with higher transcriptional activity, which was approximately 1.56-fold higher than that of the parental promoter [28]. Nowadays, on the basis of random mutagenesis in the key regions and sites within the promoter, much progress has been made in the evolution of promoter function. For example, Sauer et al. [31] developed a strategy targeting the non-conserved sequences within the *B. subtilis* native promoter (P_veg_) by designing and constructing a large combinatorial library, which led to a synthetic promoter to drive the GFP expression increased by 13-fold in comparison with the native promoter P_veg_.

*B. pumilus* is closer to *B. subtilis* in genetic background [32] and has been applied to various aspects of biotechnology such as production of industrial enzymes and secondary metabolites [33,34,35]. Previously, transcriptomic profiling at different growth phases of *B. pumilus* BA06 was analyzed to reveal that some genes were expressed at extra-high level in terms of the FPKM (Fragments per Kilobase Million) value [36]. Accordingly, transcriptome-based strategy and promoter engineering were employed to screen highly strong promoters used in recombinant protein expression of *B. subtilis* and its relatives. Finally, IPTG-inducible gene expression system based on two promoters of P_2069_ and P_2069M_ were obtained, which provides us with an alternative genetic tool for *Bacillus* species.

## 2. Materials and Methods

### 2.1. Bacterial Strains and Growth Conditions

The bacterial strains used are present in Table 1. *Escherichia coli* DH5α was used for the cloning experiment. *Bacillus subtilis* WB600 [37] and *B. pumilus* BA06 were used for recombinant expression. The strains were routinely cultured in Luria–Bertani (LB) medium (10-g/L tryptone, 5-g/L yeast extract, 10-g/L NaCl, pH 7.5) at 37 °C. If needed, LB medium was supplemented with 100-μg/mL ampicillin (Amp) for *E. coli*, 20-μg/mL kanamycin (Km) or 20-μg/mL chloramphenicol (Cm) for *B. subtilis* and *B. pumilus*.

### 2.2. Construction of B. subtilis Expression Vectors

All of the vectors used in this study are listed in Table 1. The primers used to construct each vector were listed in Table S1. The vector pSU03-AP allows to secretory expression of the alkaline protease (AprE) from *B. pumilus* BA06 in *Bacillus* [38]. pMUTIN4 was used to provide the *lac*I gene [39]. pGEM-T-HSA (Catalog No. HG10968-G) were purchased from Sino Biologic, Inc. (Beijing, China), which hosted the full cDNA encoding the human serum albumin (HSA).

In order to screen out strong promoters, 14 gene promoters were selected based on their transcriptional levels in *B. pumilus* BA06 (Table S2). The promoter sequences were amplified by PCR with 2× Phanta Max Master Mix (Vazyme Biotech Co., Ltd., Nanjing, China) and the corresponding primers (Table S1) and using genomic DNA of *B. pumilus* BA06 as template. Meanwhile, the vector skeleton was also amplified using pSU03-AP as template with the primers (P18M13-R/PAPSta-F). The PCR cycles included an initial denaturation at 95 °C for 3 min, followed by 30 cycles at 95 °C for 15 s, 56 °C for 15 s and 72 °C for 30 s to 5 min dependent on the target length and a final extension at 72 °C for 5 min. Then, both the vector DNA fragment and promoter fragments were purified with Gel Recovery Kit (Omega Bio-tek, Inc., Norcross, GA, USA). The recombinant cloning method was used to clone the promoter fragment into the vector pSU03-AP with the overlapped primers at 5′-end (Figure S1). The recombination reaction was performed following the guide of the Clonexpress^®^ II One Step Cloning Kit (Vazyme Biotech Co., Ltd., Nanjing, China). The recombinant product was directly transformed into *E. coli* DH5α. Hence, the expression vectors named as pSU03-P_xxxx_-AP were successfully obtained after confirmed by DNA sequencing.

To replace the *aprE* gene in pSU03-P_xxxx_-AP, the green fluorescent protein (GFP) gene and human serum albumin (HSA) cDNA were amplified by PCR using primers (PGFP.F/PGFP.R, PHSA.F/PHSA.R), respectively. The vector fragment was also amplified by using primers (PdeAP-F/PdeAP-R) and pSU03-P_2069_-AP as template. The recombination cloning was performed as described above, resulting in pSU03-P_2069_-GFP and pSU03-P_2069_-HSA.

To compare promoter activity of P_2069_ with the promoter P_43_ which has been recognized as a strong promoter commonly used in the recombinant expression system of *B. subtilis* [6], the P_43_ promoter sequence was amplified using the genomic DNA of *B. subtilis* WB600 as template and with the primers (P43.F/P43.R) and inserted into pSU03-P2069-GFP to replace P2069 sequence to generate the vector pSU03-P_43_-GFP by the recombinant cloning as described above.

Inducible and strong promoter is routinely applied to recombinant protein expression. Therefore, *lac*O operon sequence from the plasmid pMUTIN4 [39] was inserted between −10 box and the RBS in the promoter sequence of P_2069_ and P_2069M_ by overlapped PCR using primers (I2069-F_2_/I2069-R_2_), respectively (Figure S2A). The PCR reaction contained an initial denaturation at 95 °C for 3 min, 30 cycles with denaturation at 95 °C for 15 s, annealing at 58 °C for 15 s, extension at 72 °C for 5 min. Meanwhile, the *lac*I gene with the promoter (P*_enP_*) and the terminator sequence from the plasmid pMUTIN4 [38] were also amplified using two pairs of primers (PlacI.F/PlacI.R and PrrnBT-F/PrrnBT-R), respectively. These two fragments were subsequently cloned between the *Amp* and *Kan*-resistant genes in pSU03-P_2069_-GFP and pSU03-P_2069M_-GFP via recombination cloning (Figure S2B), respectively, resulting in two expression vectors of pSU03-P_2069_-lacI-GFP and pSU03-P_2069M_-lacI-GFP.

All of vectors that were constructed in this work were confirmed by DNA sequencing (Tsingke Biotech Co., Ltd., Chengdu, China).

### 2.3. Engineering Promoter P_2069_

Saturation mutagenesis approach was carried out to engineer the promoter P_2069_. A pair of mutagenesis primers (Prapmut-F/Prapmut-R) was designed to mutate two regions between −35 and −10 regions and −10 region and the ribosome binding site (RBS) of P_2069_, respectively. The mutagenesis PCR was performed using pSU03-P_2069_-GFP as template in 50 μL mixture containing 25 μL 2× Phanta Max Master Mix, 2 μL forward and reverse primer and about 100 ng template DNA. The PCR cycles included an initial denaturation at 95 °C for 3 min, 30 cycles of 95 °C for 15 s, 58 °C for 15 s and 72 °C for 5 min and an extension at 72 °C for 10 min. The PCR mixtures were treated with *Dpn*I to remove the template DNA and then transformed into *E. coli* competent cells as described by Sambrook et al. [40]. All the *E. coli* transformants were collected from the LB agar plates and mixed plasmid DNAs were isolated as the promoter mutation library after examination of the mutation effect by DNA sequencing.

### 2.4. Transformation of B. subtilis by Chemical Competence

The competent cells of *B. subtilis* WB600 were prepared as following: 0.3 mL of overnight culture in LB medium was transferred into 10 mL of LB medium and incubated at 37 °C up to OD_600_ to about 1.0; 0.3 mL in LB broth was transferred into 10 mL MD medium [0.92 mL 10× PC buffer (600-mM K_2_HPO_4_, 440-mM KH_2_PO_4_, 30-mM C_6_H_5_Na_3_O_7_)], 0.1 mL 5-mg/mL L-Trp, 0.1 mL 50% glucose, 0.005 mL 2.2-mg/mL ferric ammonium citrate, 0.24 mL 0.5-M potassium aspartate, 0.03 mL 1-M MgSO_4_, 8.625 mL ddH_2_O) and incubated at 37 °C till OD_600_ to about 1.0. Then, 3 mL competent cells were mixed with 1.0 μg vector DNA in a test tube and further cultivated at 37 °C for 1 h. To continue cultivation, 300 μL 10-times LB medium was added. After incubating at 37 °C with vigor shaking for another 1.5 h, the cells were harvested and plated on LB-agar plates with appropriate antibiotic.

### 2.5. Transformation of B. pumilus by Electroporation

The transformation of *B. pumilus* BA06 was carried out with reference to the high-osmolarity electroporation protocol for *B. subtilis* with some modifications [41]. The competent cells of *B. pumilus* BA06 were prepared as following: 0.5 mL of overnight culture in LB broth was transferred into 50 mL of LB medium (containing 0.5-M sorbitol and 5% betaine) and incubated at 37 °C up to OD_600_ to about 1.0; the cells were harvested by centrifugation at 5000 rpm and 4 °C for 10 min. After three washes with 50 mL of ice-cold washing buffer (0.5-M sorbitol, 0.5-M mannitol, 10% glycerol and 7.5% betaine), the cells were resuspended in 1 mL electroporation buffer (0.5-M sorbitol, 1-M mannitol, 10% Glycerol and 7.5% betaine). In the electroporation trials, 80 μL competent cells were mixed with 1 μg plasmid DNA in EP tube and transferred to the precooled L mm electroporation cuvette. Put it into the MicroPulser II (Bio-Rad Co., Hercules, CA, USA) to shock, parameter setting: 2200 V, 25 UF, 200 Ω. Immediately following the electrical discharge, 1 mL recovery medium (LB containing 0.5-M sorbitol and 0.38-M mannitol) were added to the cells. The cells were incubated overnight at 37 °C and spread on LB agar plate with appropriate antibiotics.

### 2.6. Recombinant Protein Expression and Analysis

An overnight culture of the *B. subtilis* recombinants hosting the designated vectors was inoculated into fresh LB medium containing appropriate antibiotics and incubated at 37 °C by shaking at 200 rpm. At the indicated time points, 1 mL culture was sampled from each flask. The cell density was measured at OD_600_. The extracellular alkaline protease activity was determined using casein as substrate by the Folin–Ciocâlteu method as described before [42]. The caseinolytic reaction was performed at 50 °C for exact 10 min. One activity unit was defined as the enzyme required to release 1 μg tyrosine.

To determine GFP fluorescence intensity, a single colony of the bacterial recombinant containing GFP reporter gene was transferred into 5 mL LB broth and incubated overnight at 37 °C. Then, 0.2 mL culture was transferred into 20 mL of LB in 100-mL flasks containing appropriate antibiotics, which was incubated at 37 °C by shaking at 200 rpm. The cell suspension (100 μL) was sampled at the indicated time points and loaded onto the black OptiPlate-96 F plate (PerkinElmer Co., Ltd., Waltham, MA, USA). The fluorescence intensity (expressed as a.u.) was measured with excitation at 484 and emission at 507 nm using a Synergy H1 microplate reader (BioTek, Winooski, VT, USA). The GFP expression was presented as the fluorescence intensity per OD_600_ cells.

For analyzing HSA expression, western blotting analysis was performed. An overnight culture of the *B. subtilis* recombinants carrying *HSA* reporter gene was inoculated into 20 mL fresh TSB (17.0 g tryptone, 3.0 g Soybean peptone, 2.5 g C_6_H_12_O_6_, 5.0 g NaCl, 2.5 g K_2_HPO_4_) containing appropriate antibiotics and shock at 37 °C for 24 h. The culture was harvested by centrifugation at 5000 *g* for 10 min, then washed with PBS (137-mM NaCl, 2.7-mM KCl, 10-mM Na_2_HPO_4_, 2-mM KH_2_PO_4_, pH 7.4). The cell pellet was resuspended in TSE buffer (10-mM Tris-HCl, pH 8.0, 50-mM NaCl, 1-mM EDTA) containing 100-mg/mL lysozyme and 2-mM PMSF and incubated at 37 °C for 30 min. The cells were disrupted by sonication and the resulting cellular lysate was clarified by centrifugation at 12,000 *g* for 10 min at 4 °C. Equal amount of protein samples were separated on 10% SDS-PAGE and transferred onto the PVDF membrane. A rabbit polyclonal anti-HSA antibody (Proteintech Biology Co., Ltd., Wuhan, China) was used to identify expression of HSA. The secondary donkey antibody (IgG) coagulated with Alexa Fluor^®^ 680 (Abcam Trading Co., Ltd., Shanghai, China) was used to detect the primary antibody. The signal of HSA was imaged by Azure C500 instrument (Azure Biosystems, Inc., Dublin, CA, USA).

### 2.7. Statistical Analysis

All of the above experiments were performed in triple. Moreover, the data were represented as an average value with standard deviation. The statistically significant differences between the control and the test group were calculated by two-tailed unpaired Student’s *t*-test using Microsoft Excel. The significant levels were defined with a star for *p* < 0.05, two stars for *p* < 0.01, three stars for *p* < 0.001 and four stars for *p* < 0.0001.

## 3. Results

### 3.1. Selection and Evaluation of the Promoter Strength in B. subtilis

Previously, transcriptome profiling of *B. pumilus* BA06 was carried out by RNA-seq, which showed more than 96% genes had FPKM values less than 2000. In contrast, a few of genes with extra-high FPKM values (Table S3), suggesting their expression may be controlled under strong promoters. Therefore, 14 genes (cds0544, cds0765, cds0780, cds0812, cds0828, cds1073, cds1075, cds1196, cds1571, cds1927, cds2069, cds2659, cds2646, cds3514) were selected according to their transcriptional levels (Table S2) and used to evaluate their promoter activity in the multiple copy vector in the heterologous *B. subtilis* cells.

**Figure 1:**
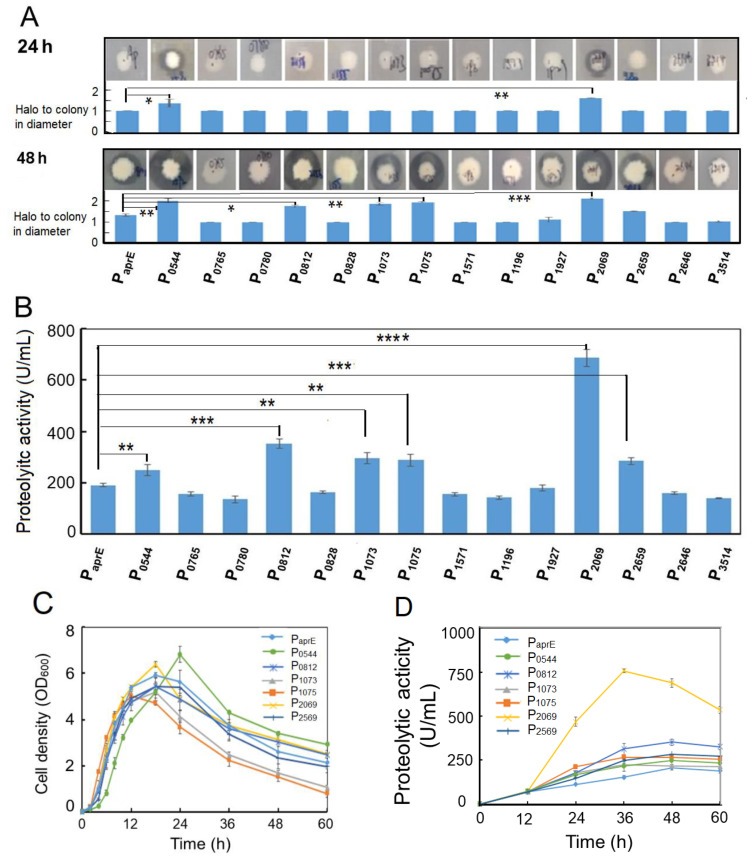

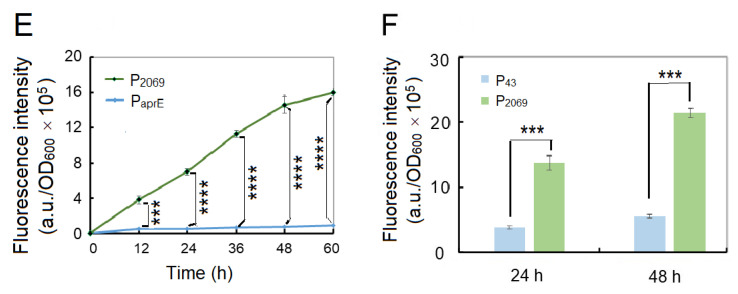
Experimental evaluation of the selected promoters from *B. pumilus* in *B. subtilis*. (**A**) Alkaline protease expression under control by different promoters was assessed by observing size of the hydrolytic circle on the milk-containing agar plates; (**B**) extracellular alkaline protease activity driven by different promoters at 48 h fermentation; (**C**) comparison of the growth pattern of the *B. subtilis* recombinants hosting the selected promoters. The bacterial strains were grown in the LB medium at 37 °C with shaking at 200 rpm; (**D**) time course of the extracellular alkaline protease production in various *B. subtilis* recombinants hosting the selected promoters. The bacterial strains were grown in the LB medium at 37 °C with shaking at 200 rpm; (**E**) fluorescent intensity and cellular growth of two *B. subtilis* recombinants hosting promoter P_2069_ or P_aprE_; (**F**) comparison of the promoter strength of P_2069_ and P_43_ in *B. subtilis.* The native promoter of the alkaline protease gene was served as the control for Figure 1A–E. Data are averages of three independent experiments with standard deviation. The significant levels were defined with “*” for *p* < 0.05, “**” for *p* < 0.01, “***” for *p* < 0.001 and “****” for *p* < 0.0001.

To assess the activity of 14 promoters, their upstream sequences (about 500–600 bp) from the start codon were amplified by PCR and inserted at the immediately upstream of the *aprE* gene encoding the alkaline protease from *B. pumilus* BA06 in the expression vector pSU03-AP [38]. The resulting 14 expression vectors were transformed into *B. subtilis* WB600 and confirmed by colony PCR. Initially, the *B. subtilis* recombinants hosting different promoters were simultaneously spotted on LB-agar plate containing 1% milk powder. By observing the size of hydrolytic circle around the colony, promoter activity to drive the alkaline protease expression could be preliminarily evaluated. Figure 1A indicated that the native promoter of *aprE* (positive control) resulted in no proteolytic circle at 24 h. In contrast, the obvious hydrolytic activity could be observed at the same time point for the promoter of cds2069 and cds0544. At 48 h, six recombinant strains (hosting promoter of cds0544, cds0812, cds1073, cds1075, cds2069 and cds2659) produced significantly larger hydrolytic circle than that of the control.

In order to confirm the highly efficient promoters, the caseinolytic activities of the 15 recombinants were determined at 48 h after fermentation. Figure 1B shows that promoter activity of the genes cds0544, cds0812, cds1073, cds1075, cds2069 and cds2659 were significantly higher than that of the control promoter P_aprE_ at *p* < 0.01 with an increase of 30.2%, 84.7%, 55.5%, 51.2%, 260.9%, 49.1%, respectively.

We further compared the cellular growth pattern and dynamic change of the alkaline protease synthesis among the selected 6 recombinants. Figure 1C shows that the growth curves had no obvious difference for various *B. subtilis* recombinants as well as the control strain. The cell density arrived at peak at 18 h, and then declined in the later. However, the recombinant strains hosting the promoter of cds1073 and cds1075 showed a quicker fall in their cell density in the later growth phase. By contrast, the alkaline protease activity of the recombinant strains was quite different (Figure 1D). The promoter of cds2069 (P_2069_) achieved the highest proteolytic activity over the entire growth phase with a proteolytic activity of 757.6 U/mL at the peak, which was 3.65-fold higher than that of the *aprE* promoter (P_aprE_).

To simple monitoring recombinant protein production in the following experiments, we used the *GFP* gene to replace the *aprE* gene in the expression vectors. Two vectors pSU03-P_2069_-GFP and pSU03-P_aprE_-GFP were constructed and transformed into *B. subtilis* WB600. Figure 1E shows that the fluorescence intensity between these two recombinant strains was significantly different (at least *p* < 0.001) at each time-point. The promoter P_2069_ driving GFP expression began at the exponential growth phase and reached a peak at the stationary phase (Figure 1E). At 60 h, P_2069_-droven GFP expression level was 18.4-fold higher than that of P_aprE_, which indicated that the promoter P_2069_ was a good candidate to express the target gene in *B. subtilis*.

The *B. subtilis* promoter P_43_ is a well-known strong promoter that has been widely used in the recombinant expression system of *B. subtilis* [6]. Therefore, we wanted to know if the P_2069_ activity is higher or lower than that of P_43_. Hence, we constructed another expression vector pSU03-P_43_-GFP and transformed it into *B. subtilis* WB600. It was found that P_2069_ was significantly more active than P_43_ when driving GFP expression in *B. subtilis* (Figure 1F). The GFP expression level under control by P_2069_ was 3.48- and 4.48-fold more than that of P_43_ at 24 h and at 48 h, respectively. Taken together, the heterologous promoter P_2069_ was much better and could be used as strong promoter in the *B. subtilis* expression system.

### 3.2. Engineering Promoter P_2069_

**Figure 2:**
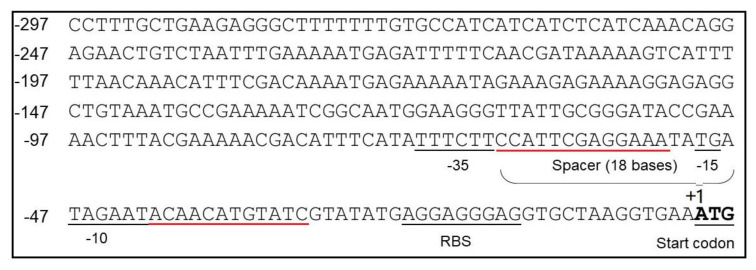
Sequence analysis of the P_2069_ promoter. The predicted −35 and −10 boxes and the spacer sequences between these two elements were indicated; The start codon and ribosome binding site (RBS) were also labeled. The regions underlined in red were selected for mutagenesis in promoter engineering.

In *B. pumilus* BA06 genome, cds2069 is annotated to encode the response regulator aspartate phosphatase A (RapA). RapA is a stage 0 sporulation protein, which is controlled by a sigma A-dependent promoter that are common in housekeeping genes and early sporulation genes in *B. subtilis* [43]. Many promoters, such as P_43_, P_yCH_ and P_secA_ controlled by sigma A-dependent promoters, exhibited strong activity during the exponential growth phase in *B. subtilis* [44,45,46]. We then analyzed the sequence of P_2069_ using software BPROM online (http://softberry.com/berry.phtmL?topic=bprom&group=programs subgroup=gfindb). Figure 2 shows the predicted −35 and −10 elements and the spacer region. However, −10 and −35 boxes are not identical with the prokaryotic conserved “TATAAT” and “TTGACA”. The distance between them is 18 bp. In addition, the conserved dinucleotide TG is found at 1 bp upstream of the −10 box.

The conservative elements of promoter, such as −10 and −35 regions, are in general important to maintain the startup function, which cannot be changed. However, the upstream and downstream regions of these conservative sequence may modulate the promoter strength [47]. Therefore, promoter engineering was performed to further enhance the P_2069_ strength by random mutagenesis of two regions between −10 and −35 regions and −10 box and the RBS, which were underlined with the red line (Figure 2). The mutagenesis PCR was performed using pSU03-P_2069_-GFP as template to generate a mutation library containing about 11,000 clones by transforming into *E. coli* DH5α. The mutation regions were confirmed by DNA sequencing.

**Figure 3:**
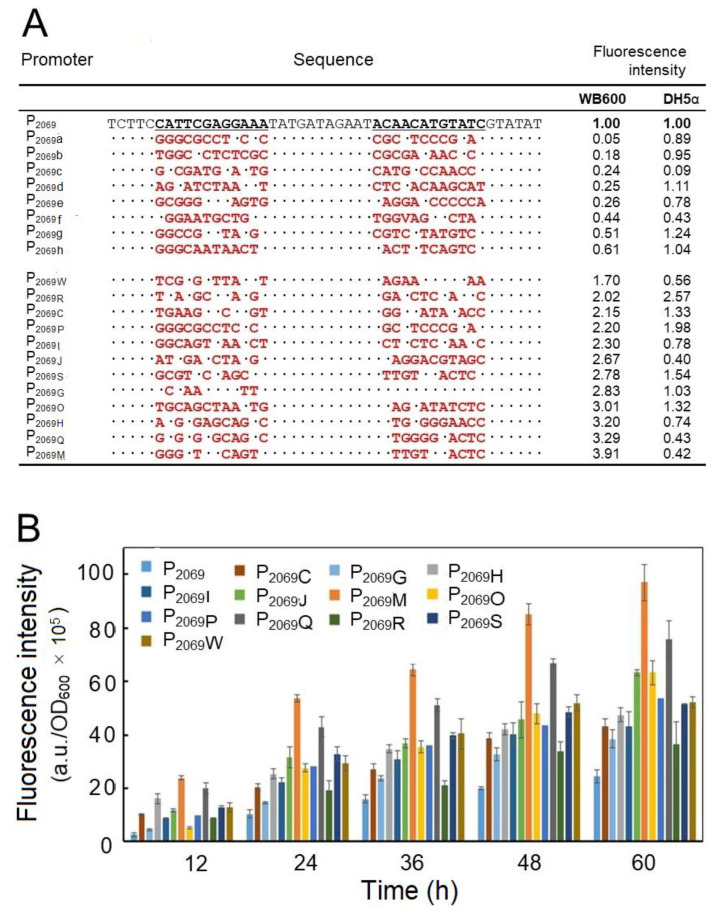
Engineering the promoter P_2069_. (**A**) DNA sequencing of the selected mutation promoters of P_2069_. The mutation promoters of P_2069_ a to P_2069_ h refer to 8 promoter variants with the reduced fluorescence intensity in comparison with the wild-type P_2069_ and P_2069 W_ to P_2069M_ refer to 12 promoter variants with the enhanced fluorescence intensity; “.” indicates no change in the nucleotide; the red letters represent the altered nucleotide; The relative fluorescence intensities against the wild-type promoter P_2069_ were measured at 24-h fermentation in *B. subtilis* and *E. coli;* (**B**) comparison of the promoter activity of the mutation promoters with the native P_2069_ in driving *GFP* expression in *B. subtilis* WB600.

The plasmid DNAs isolated from the mutation library was then transformed into *B. subtilis* WB600, and more than 10,000 transformants were obtained by several independent transformation experiments. On the LB agar plates, the transformants exhibited different fluorescent intensities, which allowed us to pick up the colonies with higher fluorescence than the control (native P_2069_ promoter). Totally, we picked up 342 clones with higher green fluorescence intensity than the wild-type promoter based on plate screening, which were then inoculated in 96-well plates for further screening through monitoring the fluorescence intensity. Finally, 23 clones with obviously increased fluorescence intensity were obtained. We recovered the plasmid DNA from the 23 clones as well as 8 clones with lower fluorescence and their nucleotide sequences were determined by DNA sequencing. By grouping the identical mutation sequences, 12 mutation promoters with increased fluorescence intensity were obtained. Figure 3A shows the altered nucleotides in the selected promoter mutants. Their promoter strengths were across a wide range over two orders of magnitude in *B. subtilis*, indicating that change of the interregions between the conservative promoter elements could produce a profound influence on the promoter activity. By the way, the promoter activity of these mutants was determined in *E. coli*, which also exhibited different strength (Figure 3A). However, the promoter activity for a given mutant was in general not consistent between *B. subtilis* and *E. coli*.

Finally, the GFP expression driven by the 12 stronger mutation promoters over 60 h-fermentation was monitored, out of which a mutation promoter designed as P_2069M_ led to the highest fluorescence intensity by 3.67-fold increase in comparison with the wild-type promoter (P_2069_) at 48 h.

### 3.3. Test of the P_2069M_ Promoter to Drive Gene Expression

**Figure 4:**
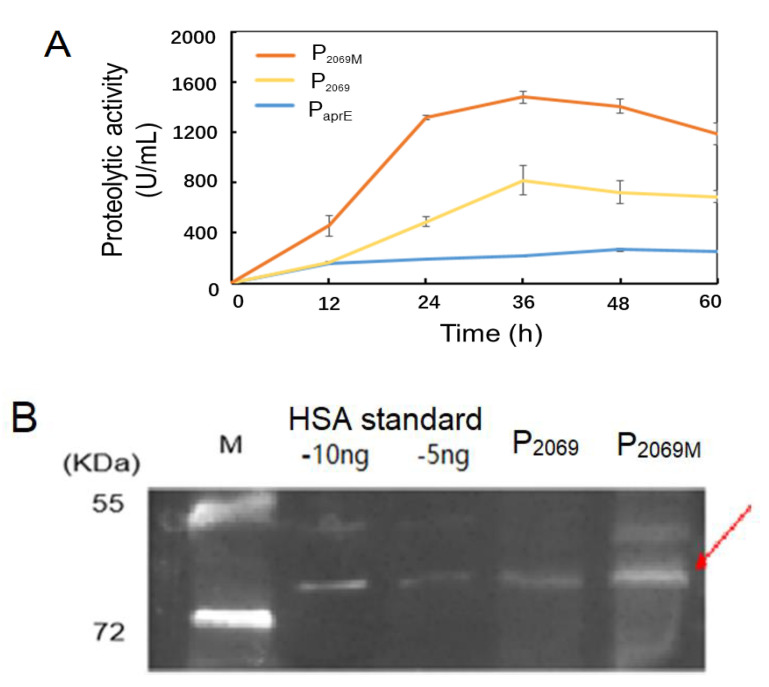
Evaluation of the mutation promoter P_2069M_ in driving expression of various heterologous genes. (**A**) Alkaline protease expression controlled by three promoters of P_aprE_, P_2069_ and P_2069M_ over 60-h fermentation; (**B**) Expression of the recombinant human serum albumin (HSA) driven by promoters P_2069_ and P_2069M_ was analyzed by the western blotting at 24 h. The arrow indicates the target protein HSA. M: Protein marker, the standard HSA loaded with 5 ng and 10 ng, respectively.

The *aprE* and HSA genes were chosen to test promoter strength of the mutation promoter P_2069M_. Two expression vectors pSU03-P_2069M_-AP and pSU03-P_2069M_-HSA were constructed and transformed into *B. subtilis* WB600. Figure 4A shows that the extracellular caseinolytic activity under control by P_2069M_ was peaked at 36 h by an increase of more than 1-fold than the wild-type P_2069_.

The *B. subtilis* recombinants carrying *HSA* gene were incubated in TSB medium for 24 h. The cellular lysates were used to detect the HSA expression level by the western blotting. Figure 4B demonstrated that the recombinant HSA protein was expressed as indicated by a protein band referring to about 66 kD. Further, HSA expression level was obvious higher with the mutant promoter (P_2069M_) in comparison with the wild-type promoter (P_2069_). These results demonstrated that the promoter P_2069M_ not only mediated the recombinant GFP expression higher, but also drove higher expression of the *aprE* and *HSA* genes in *B. subtilis*. However, increasing extent of the mutation promoter P_2069M_ is dependent on the heterologous genes by themselves.

### 3.4. Construction of Inducible Expression System in Bacillus

Inducible expression is an important strategy in recombinant protein expression, which allow us to control heterologous gene expression at certain stage, especially for production of toxic protein and achievement of high-density fermentation. Therefore, two inducible expression vectors (pSU-P_2069_-lacI-GFP and pSU-P_2069M_-lacI-GFP) were constructed by introducing *E. coli lac*O operon and *lacI* gene. These two vectors were transformed into *B. subtilis* WB600 and *B. pumilus* BA06. The resulting 4 recombinants were incubated in LB broth at 37 °C till OD_600_~2.0. Then, IPTG was added at a final concentration of 0.9 mM. After inducing expression for 4 h, the fluorescence intensities were quantified. No matter in WB600 or BA06, the fluorescence intensity increased extreme significantly in comparison with the control without IPTG addition with *p* < 0.0001 (Figure S3), indicating that our inducible system could work both in *B. subtilis* and *B. pumilus.*

**Figure 5:**
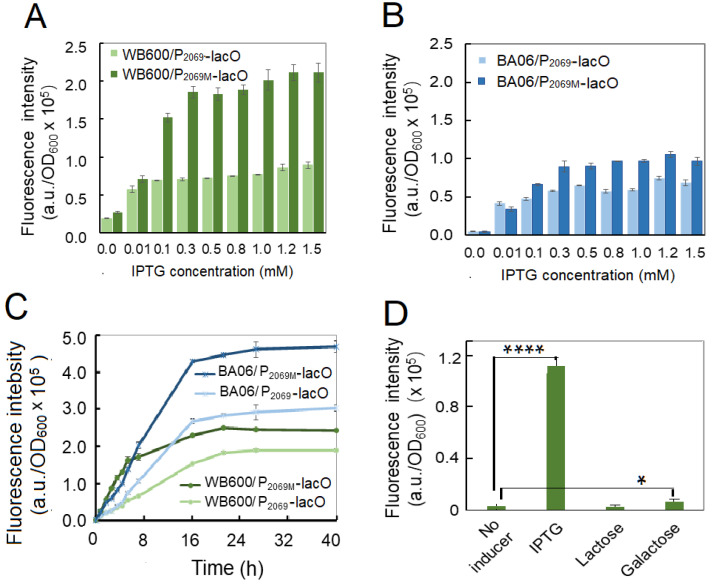
Optimization of the inducing parameters for GFP recombinant expression in *Bacillus.* (**A**) Optimization of IPTG concentrations in *B. subtilis* WB600 by induced for 4 h; (**B**) optimization of IPTG concentrations in *B. pumilus* BA06 by induced for 4 h; (**C**) optimization of inducing time in *B. subtilis* and *B. pumilus*; (**D** selection of the best inducer in *B. subtilis*.

In order to determine the optimal inducing concentration and time, various IPTG concentrations from 0.01 mM to 1.5 mM were employed, separately. Figure 5A, B shows that recombinant GFP expression was induced even at 0.01-mM IPTG both in *B. subtilis* and *B. pumilus*. However, the expression levels of GFP was quickly increased along the increase of IPTG concentration up to 0.3–0.5 mM, and then maintained steadily with the IPTG up to 1.5 mM. It was noticed that the P_2069M_-lacO was more active than the wild-type promoter under our inducing condition. Next, the inducing time was also determined from 1 h to 40 h after IPTG added at a final concentration 1.2 mM. As shown in Figure 5C, the recombinant expressed GFP was accumulated exponentially during the initial 18 h after IPTG supplemented in both strains, and then kept steadily up to 40 h. In addition, inducing expression efficiency was higher for both promoters in BA06 than in WB600, especially at the later inducing stage.

IPTG is a synthetic analog of lactose, which is usually used as an inducer in recombinant protein expression. In consideration of cost of the commercial fermentation, lactose or galactose may severed as an alternative of IPTG. Therefore, we tested if lactose and galactose could induce GFP expression in our system. Figure 5D shows lactose did not act as inducer; and galactose could induce GFP expression, but so weakly in comparison with IPTG under this condition.

## 4. Discussion

RNA sequencing technology makes the genome-wide identification of RNA-based regulatory elements with extremely low background and unprecedented depth [48]. This technology can detect a larger dynamic range of expression levels and accurately quantify transcripts with low expression levels [49]. Therefore, transcriptional analysis based on RNA sequencing is one of the most powerful tools, providing not only important insights into the functional elements of the genome, gene expression patterns and regulation, but also a simpler, more economical and effective method for applied research [50]. For example, several strong promoters were screened based on the transcriptome data and applied to recombinant protein expression in *B. subtilis* [21,51,52]. Therefore, 14 genes with extra-high expression levels (FPKM values) were selected based on the transcriptome profiling of *B. pumilus* BA06 and evaluated for their promoter strength in *B. subtilis*. As a result, a candidate promoter P_2069_ was screening out with the strongest activity, which was found to be more active than that of P_43_, a commonly used stronger promoter in the *B. subtilis* recombinant expression systems. Our results indicated that it is feasible to use the transcriptomic data even from the heterologous species to screen the strong promoter. However, the discrepancy may happen to some gene promoters. For example, the FPKM values of genes cds0765 and cds1196 were high, but their activity to drive the alkaline protease gene expression were lower. It is noticed that some of these genes such as cds0765, cds0780 are sporulation-related, which may by repressed during vegetable growth phase. In view of their performance on the multiple-copy plasmid (Figure 1B), these genes may not be suitable for recombinant protein expression. In contrast, promoter strength of the genes cds0544 with lower FPKM value achieved higher activity (Table S2). This suggests that there was no strict positive correlation between highly transcriptional level in native cells and strong promoter strength in recombinant expression. Therefore, it is necessary to confirm the promoter strength experimentally in biotechnological applications even if the promoters are derived from the highly transcribed genes.

Promoter engineering is one of the most important strategies for improving the efficiency and productivity of exogenous protein [17]. By doing so, random mutagenesis is an effective method to enhance promoter strength without explicit requirement of extensive knowledge of sequence-to-function mapping. For example, error-prone PCR (ep-PCR) can target both the consensus and non-consensus promoter regions indiscriminately. It is easy to obtain a large dynamic range of promoter function libraries [53], by which a mutation library of the bacteriophage-derived promoter P_L-λ_ was constructed and showed the mutation promoters with a 196-fold dynamic range of expression in *E. coli* [54]. However, ep-PCR may yield a large number of inactive mutants perhaps due to mutagenesis of critical elements for transcription [17]. In order to solve the limitation of large inactive pools, more targeted methods that utilize molecular understanding of promoter function can be employed. For example, a saturation mutagenesis method was used to specify the spacer region between the consensus −35 and −10 motifs [55]. A randomized linker region between −35 and −10 motifs was generated, which produced a promoter library with a 400-fold dynamic range in *Lactococcus lactis* [46]. In this study, we have constructed a mutation library of P_2069_ by saturation mutagenesis to target two regions between −35 and −10 box and −10 box and the RBS, respectively. Moreover, several mutation promoters were obtained to turn out quite strong. In contrast, some others led to the GFP fluorescence intensity decreased or even disappeared. These results indicate that the spacer context between the consensus sequences could play an important role in modulating the strength of a promoter in prokaryote. Figure 3A shows the mutation sequences of 12 promoter variants with enhanced fluorescence intensity and eight mutation promoters with weaker fluorescence intensity. It was shown that the mutation nucleotides and positions in the promoter variants were quite different. Due to the small number of sequenced mutagenesis promoters, it is difficult to reliably infer the sites or nucleotide changes to have a positive or negative impact on promoter activity.

The promoter P_2069_ controls the expression of RapA protein in *B. pumilus* BA06, which is a 0-stage sporogenesis protein. Based on the sequence analysis of P_2069_, it is likely a sigma A-dependent promoter (Figure 2). In addition, sigma A-dependent promoters occur commonly in housekeeping genes and early sporogenesis genes in *B. subtilis* [43]. Since the sigma A-dependent promoter of *B. subtilis* can also be recognized by sigma70 in *E. coli* [56]. Therefore, we also analyzed the activity of these mutation promoters in *E. coli* (Figure 3A). Compared with that in *B. subtilis*, the GFP fluorescence intensity in *E. coli* was much lower, possibly due to difference of the sigma factor-RNA polymerase complexes in the two microorganisms that recognize the promoters with different structures [57]. Therefore, some strong promoters in *Bacillus* may be relatively weak in *E. coli*. For example, P_2069M_, the strongest promoter screened from *B. subtilis*, is relatively weaker in *E. coli*.

The inducible strong promoter is an important factor to achieve high level expression of target gene in *B. subtilis*. The inducible systems take the advantage that gene expression can be switched on at the proper time during fermentation [58], which is sometimes essential, for example, when the product is toxic to the host cell. Therefore, this work is also devoted to construction of inducible expression system based on our strong promoter. It was shown that our inducting systems worked well in both *B. subtilis* WB600 and *B. pumilus* BA06. However, the inducing effect in BA06 is better than that in WB600, especially at the later inducing time as shown in Figure 5C. This may reflect the fact that the promoter P_2069_ is come from *B. pumilus*. It is noticeable that the GFP expression under the control by both promoters P_2069-lacO_ and P_2069M-lacO_ was significantly decreased comparative with the native P_2069_ and its mutation P_2069M_, which may be contributed to the increased length of the spacer region between the −10 box and the RBS in the promoters. Moreover, addition of IPTG may be another reason because IPTG seemed to reduce cellular growth of the bacteria.

## 5. Conclusions

In this study, we screened out a strong promoter P_2069_ from *B. pumilus* based on transcriptome data and experimental evaluation, which could drive foreign genes to achieve higher recombinant expression in *B. subtilis*. Further, a mutation promoter P_2069M_ was obtained by engineering the wildtype promoter with saturation mutagenesis to target two spacer regions between the −35 and −10 boxes and −10 box and the RBS, respectively. The expression level of GFP under control by P_2069M_ was further increased by 3.67-fold in comparison with P_2069_. Moreover, the IPTG-inducible expression systems were also constructed based on the strong promoter P_2069_ and P_2069M_. The expression systems constructed here are not only working well in *B. subtilis*, but also provide genetic manipulation tool for the non-model Gram-positive *B. pumilus*. Conclusively, combination of transcriptome-driven strategy and promoter engineering is a practical approach to select stronger promoters for biotechnological application and biosynthetic biology.

## Figures and Tables

**Table 1:** Bacterial strains and plasmids used in this work.

Strains and Plasmids	Description	Resource
**Strains**		
*E. coli* DH5α	*SupE44, h* *sdR17, recA1, endA1, gyrA96, thi, relA1*	Vazyme Biotech Co.
*B. subtilis* WB600	Expression host deficient in six exoprotease genes, ∆*npr*E, ∆*apr*A, ∆*epr,* ∆*bpf,* ∆*nprB*, ∆*mpr*::*cat*^R^	[37]
*B. pumilus* BA06	Wide-type strain, producing alkaline protease	This laboratory
**Plasmids**		
pSU03-AP	*Amp*^r^, *kan*^r^, *ori*, *DSO*, *SSO*, *rep*, *aprE*	[38]
pMUTIN4	*Amp*^r^*lacZ ori lac*I	[39]
pGEM-T-HSA	*Amp*^r^, *ori, f1ori, HSA*	Sino Biologic, Inc.
pSU03-P_xxxx_-AP *	*Amp*^r^, *kan*^r^, *ori*, *DSO*, *SSO*, *rep*, *aprE*	This work
pSU03-P*_AprE_*-GFP	*Amp^r^ lacZ kan^r^ ori DSO SSO rep GFP*	This work
pSU03-P_2069_-GFP	*Amp^r^ lacZ kan^r^ ori DSO SSO rep GFP*	This work
pSU03-P_2069X_-GFP **	*Amp^r^ lacZ kan^r^ ori DSO SSO rep GFP*	This work
pSU03-P_2069M_-GFP	*Amp^r^ lacZ kan^r^ ori DSO SSO rep GFP*	This work
pSU03-P_43_-GFP	*Amp^r^ lacZ kan^r^ ori DSO SSO rep GFP*	This work
pSU03-P_2069_-lacI-GFP	*Amp^r^ lacZ kan^r^ ori DSO SSO lacI rep GFP*	This work
pSU03-P_2069M_-lacI-GFP	*Amp^r^ lacZ kan^r^ ori DSO SSO lacI rep GFP*	This work
pSU03-P_2069_-AP	*Amp^r^ lacZ kan^r^ ori DSO SSO rep DHAP*	This work
pSU03-P_2069M_-AP	*Amp^r^ lacZ kan^r^ ori DSO SSO rep DHAP*	This work
pSU03-P_2069_-HSA	*Amp^r^ lacZ kan^r^ ori DSO SSO rep HSA*	This work
pSU03-P_2069M_-HSA	*Amp^r^ lacZ kan^r^ ori DSO SSO rep HSA*	This work

*: P_xxxx_ represents the promoter of different genes; **: P_2069_X represents the mutation promoter derived from P_2069_. *cat*^r^*,* chloramphenicol-resistant gene*; Amp^r^*: ampicillin-resistant gene; *kan*^r^, kanamycin-resistant gene; *rep*, replication initiation gene; *SSO*, single-strand origin; *DSO*, double strand origin.

## Supplementary Materials

The following are available online at https://www.mdpi.com/2076-2607/8/7/1030/s1. Table S1: Primers used to construct the expression vectors and for DNA sequencing; Table S2: Expression levels (FPKM) of the selected genes in *B. pumilus* BA06; Table S3: Grouped statistics of the gene numbers with various transcriptional levels in the transcriptome profiling of *B. pumilus* BA06; Figure S1: Scheme to construct expression vectors via the recombinant cloning; Figure S2: Construction of the inducible expression vectors with the *lacO* system; Figure S3: Inducing expression of recombinant GFP in *B. subtilis* WB600 and *B. pumilus* BA06.
