# Supplementary material for: Construction of a High-Expression System in Bacillus through Transcriptomic Profiling and Promoter Engineering
Source: Microorganisms. 2020 Jul 12;8(7):1030. doi: 10.3390/microorganisms8071030 (PMC7409208; doi:10.3390/microorganisms8071030)
Supplement: Supplementary file 1 [file microorganisms-08-01030-s001.zip › Supplementary_Figure S1-3.pdf]

Supplementary Figure S1-3

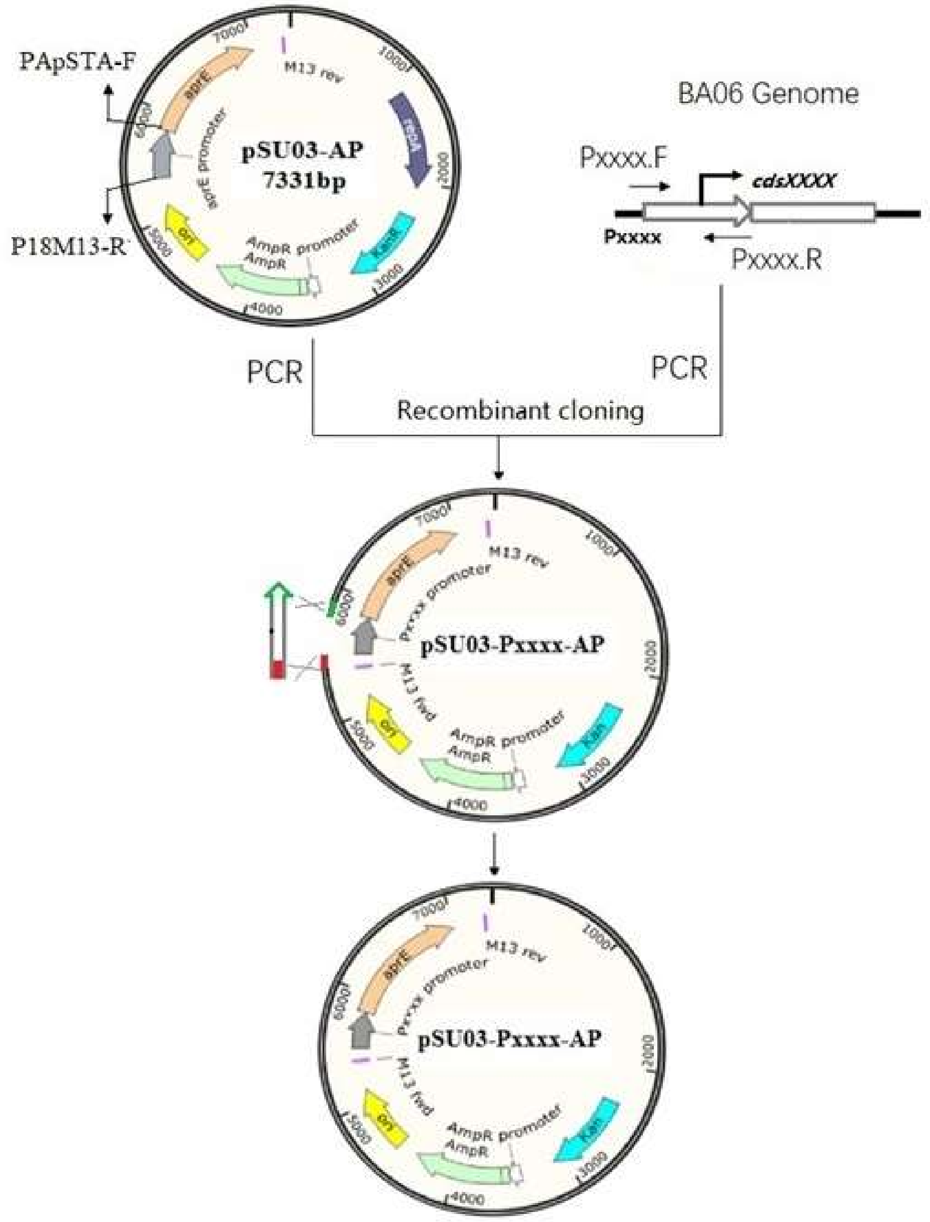

Figure S1. Scheme to construct expression vectors via the recombinant cloning. The vector skeleton was first amplified using pSU03-AP as template with the specific primers. Each of the promoter sequence was amplified by PCR using genomic DNA of *B. pumilus* BA06 as template and the specific primers. The resulting two DNA fragments were recombined by the recombinant cloning method with the overlapped sequences at both ends of the DNA fragments. The recombinant products were directly transformed into *E. coli* DH5α, and the expression vectors named as pSU03-P$_{xxxx}$-AP were obtained after confirmed by DNA sequencing.

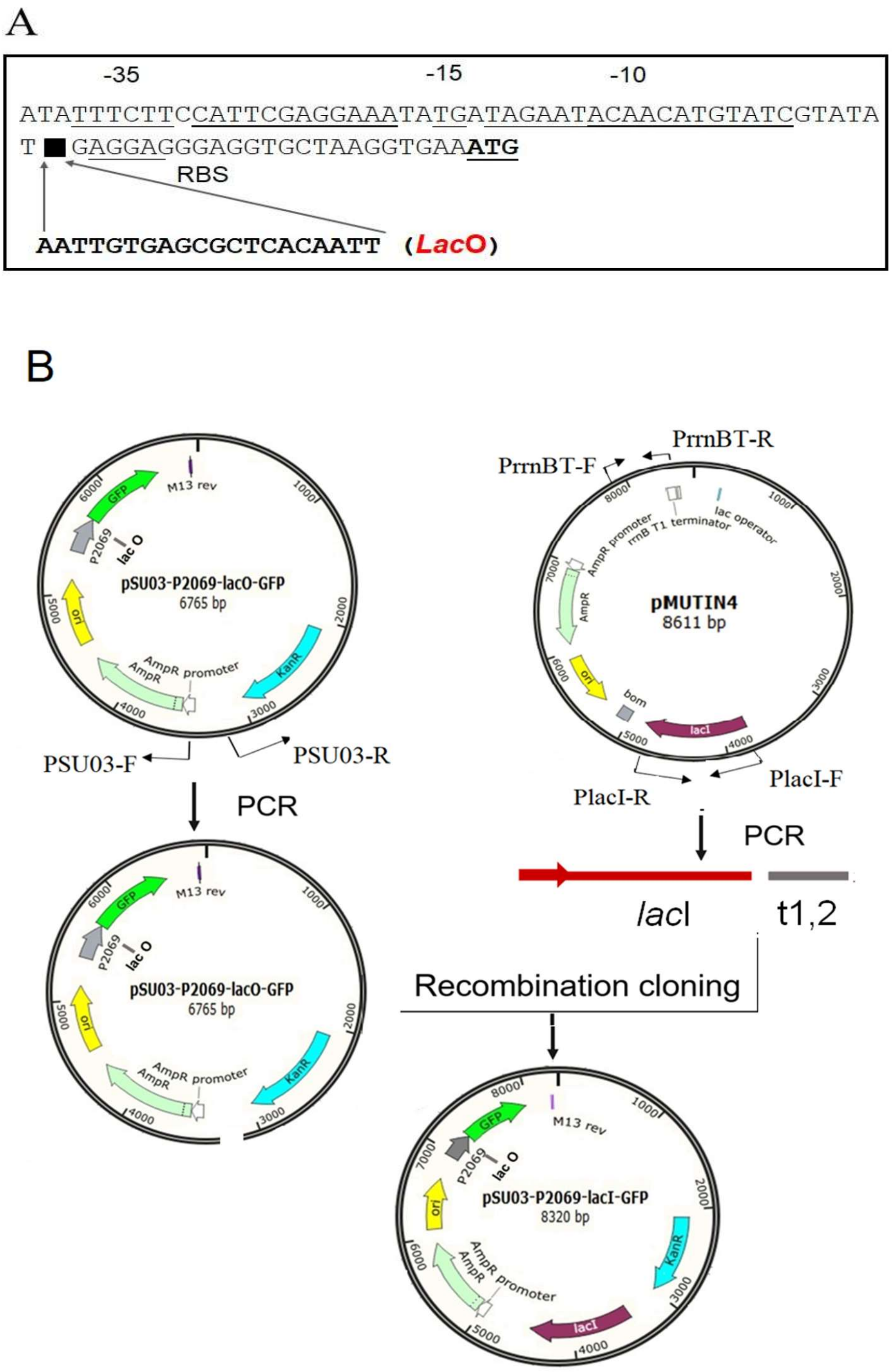

Figure S2. Construction of the inducible expression vectors with the *lacO* system. (A) The *lac*O operon sequence was inserted between the RBS and -10 box in the promoter sequence of P_{2069} and P_{2069}M by overlapped PCR with the primers (I2069-F2: 5′-TTGTGAGCGGATAACAA TTAAACGACATTTCATATTTCTTCC-3′; I2069-R2: 5′-TTGTTATCCGCTCACAATTTTCGT AAAGTTTTCGGTATCC-3′), respectively. (B) The *lac*I gene with the promoter from pMUTIN4 was first amplified by PCR and inserted between the into the *Amp* and *Kan* resistant genes in the vectors of pSU03-P_{2069}-lacO-GFP and pSU03-P_{2069}M-lacO-GFP by the recombinant cloning method, respectively; and then the terminal sequence was also amplified by PCR and inserted into the above vectors by the recombinant cloning method. Finally, two inducible expression vectors of pSU03-P_{2069}-lacI-GFP and pSU03-P_{2069}M-lacI-GFP were obtained after confirmed by DNA sequencing.

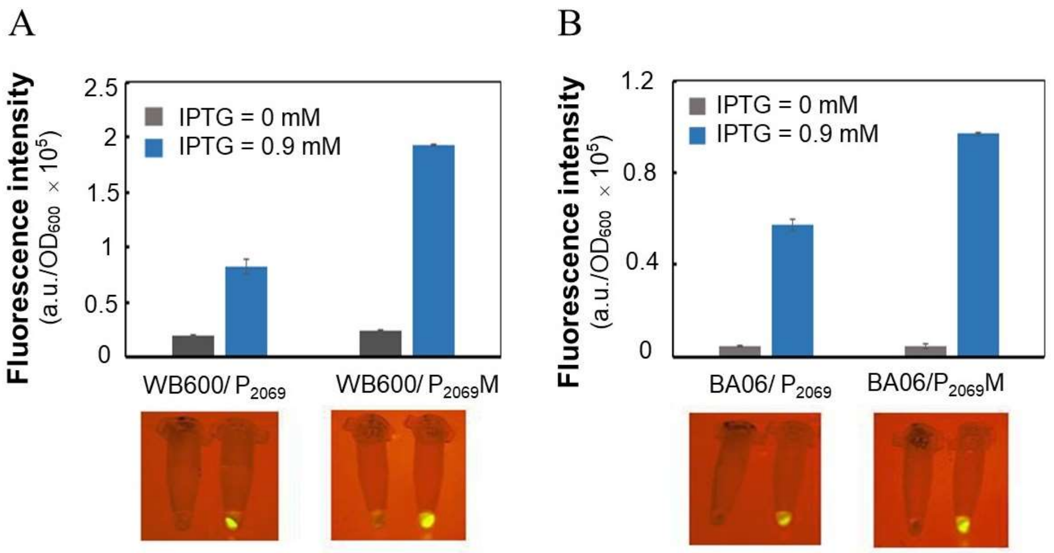

Figure S3. Inducing expression of recombinant GFP in *B. subtilis* WB600 and *B. pumilus* BA06. The fluorescence intensity was measured with excitation at 484 and emission at 507 nm using a Synergy H1 microplate reader (BioTek, Vermont, USA) (the up panel) and photographed under UV light transmission (the down panel) after 4-h induction by IPTG addition under control by the promoters of P_{2069} and P_{2069}M in *B. subtilis* WB600 (A) and *B. pumilus* BA06 (B), respectively. The bacterial cultures were grown in LB medium at 37°C and shaking at 200 rpm.
